# Use of Avian Bornavirus Isolates to Induce Proventricular Dilatation Disease in Conures

**DOI:** 10.3201/eid1603.091257

**Published:** 2010-03

**Authors:** Patricia Gray, Sharman Hoppes, Paulette Suchodolski, Negin Mirhosseini, Susan Payne, Itamar Villanueva, H.L Shivaprasad, Kirsi S. Honkavuori, Thomas Briese, Sanjay M. Reddy, Ian Tizard

**Affiliations:** Texas A&M University, College Station, Texas, USA (P. Gray, S. Hoppes, P. Suchodolski, N. Mirhosseini, S. Payne, I. Villanueva, S.M. Reddy, I. Tizard); California Animal Health and Food Safety Laboratory System, Fresno, California, USA (H.L. Shivaprasad); Columbia University, New York, New York, USA (K.S. Honkavuori, T. Briese)

**Keywords:** Avian bornavirus, viral encephalitis, proventriculus, parrots, viruses, research

## Abstract

Avian bornavirus (ABV) is a newly discovered member of the family *Bornaviridae* that has been associated with the development of a lethal neurologic syndrome in birds, termed proventricular dilatation disease (PDD). We successfully isolated and characterized ABV from the brains of 8 birds with confirmed PDD. One isolate was passed 6 times in duck embryo fibroblasts, and the infected cells were then injected intramuscularly into 2 healthy Patagonian conures (*Cyanoliseus patagonis)*. Clinical PDD developed in both birds by 66 days postinfection. PDD was confirmed by necropsy and histopathologic examination. Reverse transcription–PCR showed that the inoculated ABV was in the brains of the 2 infected birds. A control bird that received uninfected tissue culture cells remained healthy until it was euthanized at 77 days. Necropsy and histopathologic examinations showed no abnormalities; PCR did not indicate ABV in its brain tissues.

## Abstract 2

The fulfillment of Koch’s postulates shows that the virus causes proventricular dilatation disease in parrots.

Proventricular dilatation disease (PDD) is a progressive, invariably fatal neurologic disease that has been reported for >50 species of psittacine birds as well as many other bird species (*1*). It is considered a serious disease because many of these birds are highly endangered, and several affected species depend on captive breeding for their survival. The clinical signs of PDD vary and may be predominately neurologic (weakness, ataxia, proprioceptive deficits, seizures, blindness), gastrointestinal (weight loss, passage of undigested food, regurgitation, delayed crop emptying), or a combination thereof (*2*). The gastrointestinal signs, especially proventricular dilatation, are secondary to pseudo-obstruction brought about by damage to the enteric nervous system. PDD is characterized by severe lymphoplasmacytic inflammation in peripheral, central, and autonomic nervous tissues (*3*–*5*). Definitive diagnosis of PDD requires demonstration of lymphoplasmacytic ganglioneuritis in the intestinal tract.

Recently, 2 independent groups of investigators identified a new member of the family *Bornaviridae*, named avian bornavirus (ABV), in parrots with histopathologically confirmed PDD. Honkavuori et al. used unbiased high-throughput sequencing to identify the virus in several parrots with histopathologically confirmed PDD (*6*). Quantitative PCR confirmed the presence of the virus in brain, proventriculus, and adrenal gland in 3 birds with PDD but not in 4 unaffected birds. Kistler (*7*) used a panviral microarray to identify a bornavirus hybridization signature in 5 of 8 birds with PDD and 0 of 8 controls. These investigators used ultra high-throughput sequencing combined with conventional PCR-based cloning to recover a complete viral genome sequence. Before this discovery, the family *Bornaviridae* contained only 1 species, *Borna disease virus* (BDV). BDV causes a neurologic syndrome, Borna disease, which is restricted to central Europe, where it is found primarily in horses and sheep. The virus infects neurons and astrocytes, and the resulting disease appears to be mediated by an immunopathologic response of the host to the virus.

BDV can be grown in mammalian cell culture, where it causes a noncytolytic persistent infection. Borna disease appears as a sporadic infection affecting small numbers of animals each year. Its epidemiology is unclear, but it may be carried by certain species of shrews (*8*). BDV has also been detected in the feces of wild birds and in captive ostriches, but the epidemiologic significance of this observation is unclear (*9*,*10*). Studies undertaken in this laboratory have demonstrated some histopathologic similarities, in particular in the selective destruction of cerebellar Purkinje cells, between ABV and BDV infections of the brains of birds and mammals, respectively (*11*).

Seven ABV genotypes have been identified based on partial genome sequencing (*12*,*13*). In general, these ABV strains show only ≈65% sequence identity with BDV. Nevertheless, the overall structure of the bornaviral genome is well conserved (*6*,*7*). Thus, the number and order of genes is unchanged, as is the structure of transcription initiation and termination sites. Recently, Rinder et al. (*14*) have shown that the region between the N and X gene in ABV is shorter than that in BDV. ABV apparently lacks a 22-nt fragment that serves a regulatory function for the genes coding for viral proteins X and P.

Although these discoveries suggest that ABV is a plausible cause of PDD, as described in Koch’s postulates, proof of a causal relationship requires isolation of the agent from infected birds; its propagation in culture; and, after reintroduction of the isolate into a susceptible host, manifestation of the disease (*15*). We describe the isolation and culture of ABV from the brains of 8 psittacine birds with histopathologically confirmed PDD. After 6 passages, 1 of the cultured isolates was intramuscularly injected into 2 healthy Patagonian conures (*Cyanoliseus patagonis*). Typical PDD subsequently developed in each bird, and the inoculated virus was found in the brain.

## Materials and Methods

### Parrots

From independent sources we obtained 8 parrots that had clinical signs of PDD, were clinically judged to be in the late stages of the disease, and were euthanized for humane reasons. The 8 birds were 1 green-winged macaw (*Ara chloroptera*), 1 scarlet macaw (*A. macao*), 2 blue and yellow macaws (*A. ararauna*), 2 yellow-collared macaws (*Primolius auricollis*), 1 African gray parrot (*Psittacus erithracus*), and 1 umbrella cockatoo (*Cacatua alba*). Four parrots with conditions not related to PDD and euthanized for humane reasons were also included in the study as negative controls.

### Necropsy

Immediately after euthanasia, complete necropsies were performed on all birds. Tissue samples from brain, spinal cord, peripheral nerves, lungs, heart, liver, spleen, pancreas, adrenal glands, kidneys, crop, proventriculus, ventriculus, intestine, and cloaca were placed in 10% buffered formalin for histopathologic examination. Tissue sections were stained with hematoxylin and eosin to confirm the clinical diagnosis. Half of each brain was retained for virus isolation, Western blot, and reverse transcription–PCR (RT-PCR).

### Tissue Culture

Specific pathogen–free duck eggs were obtained from the US Department of Agriculture Avian Disease Laboratory (East Lansing, MI, USA). Embryos 9–10 days old were harvested, macerated, and cultured. Primary duck embryonic fibroblasts (DEFs) were used for virus isolation and propagation. DEFs were maintained in Leibowitz L15–McCoy 5A medium (LM; Sigma-Aldrich, St. Louis, MO, USA) supplemented with 5% bovine calf serum (Sigma-Aldrich) and 1% penicillin-streptomycin at 37°C in an atmosphere of 5% CO_2_. DEFs were seeded to confluency 24 h before inoculation. DEFs not inoculated with tissue homogenate were maintained in parallel throughout the experiment.

### Virus Isolation and Culture

Brain tissue was harvested immediately after euthanasia. Sections of the cerebrum and cerebellum were homogenized, minced, and then passed through an 18-gauge needle in LM complete medium. In instances where immediate culture inoculation was not possible, the brain tissue was frozen at –80°C within minutes of being harvested and was thawed in a 37°C water bath immediately before inoculation. One milliliter of the brain suspension was used to inject previously plated DEF monolayers that were then incubated for 24 h. The injected DEF cultures were then washed once with phosphate-buffered saline (PBS), replaced with fresh LM medium supplemented with 2% fetal calf serum, and incubated for 5–7 days. Infected DEFs were trypsinized and cocultivated with freshly plated DEFs. This procedure was repeated for a minimum of 3 passages.

### Western Blot Analysis

Infected DEFs passaged a minimum of 3 times were used for Western blot analyses. Samples from infected DEFs were collected by trypsinization and pelleted by centrifugation, and pellets resuspended in PBS were sonicated on ice at 50% intensity for 5 min (Sonifier 250; Branson Ultrasonics Corp, Danbury, CT, USA); 50% intensity). Western blotting was performed as described by Towbin et al. (*16*) by using 10% polyacrylamide gels and a Mini-Protean II gel electrophoresis apparatus (Bio-Rad, Hercules, CA, USA). The tissue culture preparations were diluted in sample loading buffer containing β-mercaptoethanol at a ratio of 1:1 and heated to 95°C for 5 min before being loaded (30 µg/slot). A prestained sodium dodecyl sulfate–polyacrylamide gel electrophoresis standard covering the 6.5- to 200-kDa range was used for molecular weight estimation (Bio-Rad).

The size-fractionated antigen preparations were transferred to Immobilon polyvinylidene fluoride transfer membranes (Millipore, Bedford, MA, USA) as described by Towbin et al. (*16*). Transfer efficiency was indicated by the presence of prestained bands on the membranes. After transfer, the membranes were incubated for 2 h in PBS, 0.05% Tween-20, 3% skimmed milk (PBST blocking buffer), then with histopathologically confirmed PDD-positive parrot serum diluted 1:5,000 in PBST blocking buffer for 2 h and with horseradish peroxidase–conjugated goat anti-macaw immunoglobulin Y (Bethyl Inc., Montgomery, TX, USA) diluted 1:10,000 in PBST blocking buffer for 1 h. Membranes were washed with PBST after each step, and all steps were performed at room temperature under constant shaking. Finally, the membranes were incubated for 30 min in Sigma-Fast 3,3′-diaminobenzidine developing substrate (Sigma-Aldrich) and then rinsed in distilled water. The serum from a confirmed PDD-positive parrot used in this experiment has been shown to contain antibodies specific for the 38-kDa ABV N-protein by its reaction with 2 preparations of recombinant protein prepared in *Escherichia coli* and in mammalian cell systems (*11*).

### Indirect Immunofluorescent Assay

Infected DEF were washed 2 times for 5 min each in 0.02M PBS, fixed for 10 min in 2% paraformaldehyde in 0.02 M PBS, and washed 2 times for 5 min each in 0.02 M PBS. Cells were permeabilized in 1% Triton X-100/0.02 M PBS for 10 min and washed 3 times for 5 min each in 0.3% Tween/0.02 M PBS. Blocking was performed for 2 h in 5% dried milk/0.3% Tween/0.02 M PBS. The cells were incubated in a humidified chamber for 30 min at 37°C with the primary antibody (serum from a parrot with histopathologically confirmed PDD) at a 1:500 dilution in 1% dried milk/0.3% Tween/0.02 M PBS. Cells were washed 3 times for 5 min each in 0.03% Tween/0.02 M PBS. The cultures were then incubated in a humidified chamber for 30 min at 37°C with the secondary antibody (horseradish peroxidase– or fluorescein isothiocyanate–conjugated goat anti-macaw immunoglobulin G; Bethyl Inc.) at a 1:500 dilution in 1% dried milk/0.3% Tween/0.02M PBS. Cells were washed 3 times for 5 min each in 0.03% Tween/0.02M PBS and then rinsed in distilled water and mounted with ProLong antifade reagent with DAPI (Invitrogen, Carlsbad, CA, USA).

### RT-PCR

Total RNA was isolated from collected brain tissue and passaged DEF by using the RNeasy Mini Kit (QIAGEN, Valencia, CA, USA). First-strand cDNA was generated by using the High Capacity cDNA Reverse Transcription Kit (Applied Biosystems, Foster City, CA, USA), with 1µg RNA and random primers. PCR for ABV N-protein was performed by using 1–2 µL cDNA and forward (5F: 5′-GCGGTAACAACCAACCAGCAA3-′) and reverse (1212R: 5′-GTTCATTAGTTTGCRAATCCRGTTA3-′) primers, which were developed using GenBank submissions NC_001607.1, FJ169441.1, and FJ169440.1 for reference. Amplification conditions were as follows: 1 cycle at 94°C for 2 min; 35 cycles at 94°C for 30 sec, 50°C for 30 sec, and 72°C for 80 sec; final extension at 72°C for 7 min. PCR products were cloned in TOPO-TA vector (Invitrogen), and individual clones were sequenced after transformation into *E. coli*. DNA sequencing reactions were performed by using the ABI BigDye Terminator Cycle Sequencing Kit, and sequences were generated with an ABI PRISM 3100 Genetic Analyzer (Applied Biosystems). Sequences were assembled and aligned by using Geneious Pro 4.6.2 software (www.geneious.com). Isolates were assigned to previously defined ABV groups by comparing a 397-nt region to sequences representing avian bornaviruses 1–5 (GenBank accession nos. FJ002329, FJ603688, FJ002328, FJ603687, FJ002335). Evolutionary distances were computed by using a Kimura 2-parameter model with MEGA4 software (www.megasoftware.net).

### Experimental Infections

Experimental infections were performed under Animal Use Protocol no. 2009–033B approved by the Texas A&M University Institutional Animal Care and Use Committee. Three adult Patagonian conures were shown to be seronegative by Western blotting and to be ABV negative by fecal PCR. All 3 birds were known to be chronic carriers of psittacine herpes virus; 1 had a cloacal papilloma, but all were otherwise in good health. Psittacine herpes virus has never been implicated in PDD. Two birds were placed in isolation and inoculated by intramuscular injection with infected DEFs containing 8 × 10^4^ focus-forming units (*17*) of an ABV4 (M24) originally isolated from a yellow-collared macaw. A large batch of the M24 strain was grown for 5 days on passage 6, and 500-μL aliquots of this batch were frozen at –80°C in freezing medium. Two of the 500-μL aliquots were grown for 5 days, and immunohistostaining (by using the immunofluorescent antibody [IFA] assay described, substituting the fluorescein isothiocyanate–labeled antibodies with horseradish peroxidase–labeled antibodies) was used to visualize and quantify the focus-forming units.

## Results

### Isolation and Culture of Avian Bornavirus Isolates

**Figure 1:**
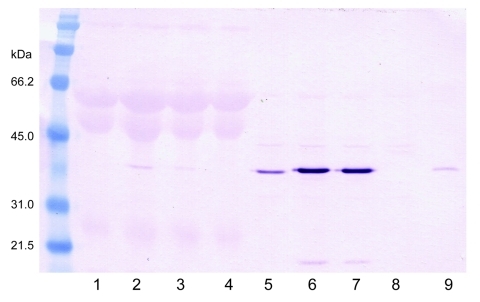
Western blot of infected duck embryonic fibroblasts (DEFs) showing avian bornavirus N-protein during culture. Lanes 1–4 are supernatant fluids. Lane I is from an African gray parrot (AG5). Lanes 2 and 3 are from a yellow-collared macaw (M24). Lane 4 is from uninfected DEFs. Lanes 5–8 are sonicated cell extracts. Lane 5 from AG5; 6 and 7 from M24; and Lane 8 from uninfected DEFs. Lane 9 is an infected brain control. The virus is strongly cell associated.

**Figure 2:**
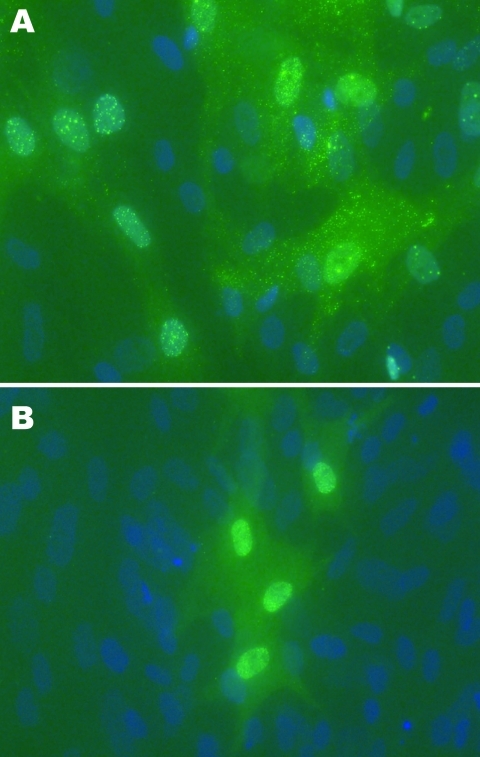
A) Avian bornavirus (ABV)–infected duck embryonic fibroblast (DEF) cell culture 6 days after injection with hindbrain tissues from an African gray parrot with confirmed proventricular dilatation disease (AG5) and staining by an indirect immunofluorescence assay for ABV N-protein. Speckled immunofluorescence is typical of bornavirus infection. Original magnification ×40. B) DEFs 3 days after injection with forebrain from a yellow-collared macaw with confirmed proventricular dilatation disease (M24). Nuclear and cytoplasmic fluorescence in DEFs stained by immunofluorescence assay for ABV N-protein. Original magnification ×40.

Cytopathic effects were not observed in any of the 12 DEF cultures inoculated with brain tissue harvested from birds with or without clinical signs of PDD. Western blot analyses showed a pronounced ABV N-protein band in extracts of 8 of the 12 cultures. Only DEF cultures inoculated with samples from parrots displaying histopathologically confirmed PDD were positive by Western blotting (Figure 1). ABV N-protein was not detectable in the 4 cultures injected with brain tissue from birds with no histologic evidence of PDD. IFA of infected DEFs also demonstrated ABV N-protein within cells. Foci of antigen-positive cells were apparent 3 days after culture inoculation. Many cells showed both nuclear and diffuse cytoplasmic staining. Other cells showed the characteristic punctate nuclear staining of infected cultures (Figure 2). No positive immunofluorescence was observed in uninfected DEFs or in DEFs inoculated with brain tissue from negative control birds.

### Characterization of Avian Bornavirus Isolates

RNA was isolated from the brain tissues and infected DEF cultures of 8 parrots with PDD and 4 parrots that were PDD negative. A 397-bp region of the ABV N-gene was amplified from all 8 PDD brain and tissue culture samples but not those from the 4 negative parrots. The amplicons were cloned and their sequences compared with previously described ABV groups (*7*). One isolate, M25, was most closely related to ABV group 1, whereas the other 7 ABV isolates were most closely related to ABV group 4 (Table). Pairwise comparisons among the group 4 isolates ranged from 94.2% to 99.7% nucleotide identity. When 2–3 complete N-protein gene sequences (1,143 nt) originating from any bird were compared, nucleotide sequence identity ranged from 99.2% to 100% (data not shown).

**Table:** Percent nucleotide identity between partial N genes in avian bornavirus isolates

Gene	ABV Type 1a*	M25	M20	AG5	M15	M10	M14	M24	06	ABV Type 4b†
ABV Type 1a	100.0									
M25	89.4	100.0								
M20	80.9	82.0	100.0							
AG5	80.9	82.0	99.7	100.0						
M15	80.9	82.0	99.9	99.7	100.0					
M10	80.9	82.0	99.6	99.5	99.7	100.0				
M14	81.1	81.7	99.6	99.6	99.7	99.6	100.0			
M24	81.1	81.7	95.0	94.8	94.7	94.5	94.5	100.0		
06	81.4	81.8	94.5	94.5	94.4	94.3	94.2	99.3	100.0	
ABV Type 4b	80.6	81.4	94.0	94.7	94.7	94.7	95.0	92.7	94.5	100.0

*ABV genotype 1 accession no. FJ002329. †ABV genotype 4 accession no. FJ603687.

### Experimental Infections

**Figure 3:**
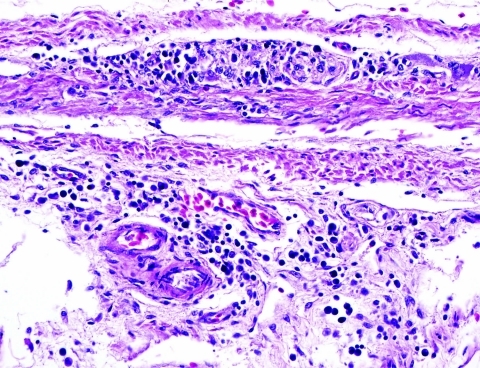
Proventriculus wall from conure PG8 showing characteristic lymphoplasmacytic infiltration of the subserosal enteric ganglia as well as infiltration of submucosa. This bird had been inoculated 55 days earlier with avian bornavirus, genotype 4. Original magnification ×325.

**Figure 4:**
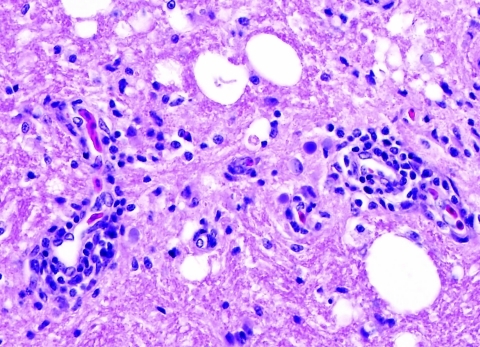
Lymphoplasmacytic encephalitis with multifocal perivascular cuffing in the cerebrum of conure PG8 inoculated 55 days earlier with avian bornavirus genotype 4. Original magnification ×725.

Two Patagonian conures were challenged with ABV4, strain M24. They were tested by fecal PCR before challenge and at 33, 43, 60, and 62 days postchallenge. Both conures were seronegative by Western blotting before challenge but seropositive for antibodies to the 38-kDa N-protein on day 33 and thereafter. Fecal PCR testing showed that both birds were negative on days 33 and 43. One bird was weakly positive on day 60, but both were strongly positive on day 62. One inoculated bird died on day 66. Necropsy showed a dilated proventriculus and gross lesions characteristic of PDD. Subsequent histopathologic examination confirmed that the bird had a lymphoplasmacytic ganglioneuritis typical of PDD in the crop, proventriculus, gizzard, and intestine (Figure 3). This gangloneuritis included mild to severe infiltration of lymphocytes and a few plasma cells in the serosa, subserosal nerves, and ganglia. The bird also had adrenalitis, encephalitis, and neuritis, as well as a myocarditis. The heart showed a lymphocytic infiltration of the epicardial ganglia as well as in and around Purkinje fibers. Thus, the brain and spinal cord showed multifocal perivascular cuffing and gliosis (Figure 4). The adrenal medulla was infiltrated with lymphocytes and plasma cells.

**Figure 5:**
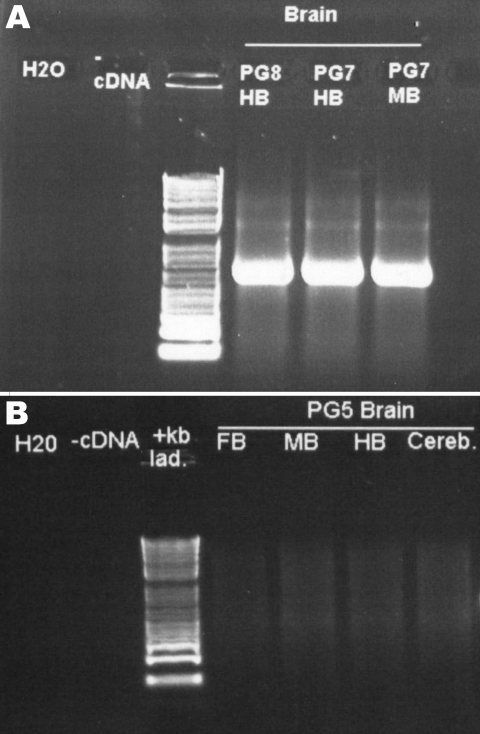
PCR of avian bornavirus N-protein in different areas of the brains of A) 2 Patagonian conures (PG7 and PG8) inoculated 55 days earlier with avian bornavirus–infected duck embryonic fibroblasts and B) control, uninfected bird, PG5. HB, hindbrain; FB, forebrain; MB, midbrain; Cereb., cerebrum.

The second inoculated bird was examined on day 66 and was found to be emaciated and had clinical signs consistent with PDD. It was euthanized for humane reasons. This bird also had gross and histopathologic lesions characteristic of PDD, essentially identical to those described above. The brains of both conures were subjected to PCR for ABV N-protein as described above. Results for both were positive (Figure 5). Sequence analysis of the PCR products confirmed that bird brains contained ABV4 identical to the M24 challenge strain. Brain homogenates from these 2 birds were also cultured on DEFs, and a strong positive PCR signal was obtained at day 16 of culture.

The third conure in this study received uninfected DEFs by both intramuscular and oral routes as described for experimentally infected birds. This bird was housed in an aviary separate from the isolation facilities of the infected birds. It was in apparent good health when euthanized on day 77. Necropsy of the bird conducted, including histopathologic examination of its tissues, and PCR was performed on 4 regions of its brain. No evidence of PDD was seen during necropsy or histopathologic examination, and all 4 brain samples were negative for ABV nucleic acid.

## Discussion

Although it has long been proposed that a viral pathogen was responsible for PDD, past attempts to identify a causal agent through inoculation of chick embryos and a variety of tissue cultures were unsuccessful. Because no cytopathic effects were detected in the DEF cultures after several passages, prior attempts to grow the agent may have been successful but had not been recognized because of lack of immunologic or PCR detection tools. We were able to isolate and propagate ABV from all studied birds with clinical PDD. IFA of infected DEF using this same antiserum showed the punctate nuclear staining that is typical of cells infected with bornaviruses and appears to be the result of the formation of N-and P-protein complexes known as Joest-Degen inclusion bodies (*18*–*20*). It is noteworthy that we were unable to grow ABV in primary chicken embryo fibroblasts handled the same as the DEFs. Rinder et al. reported successful propagation of ABV in the chicken LMH hepatoma cell line (*14*). However, they noted slow growth and only a few positive cells compared with propagation in the quail fibroblast cell line CEC32 and the quail skeletal muscle cell line QM7. Thus ABV appears to have constraints in host cell range. PDD in chickens has not been reported. Rinder et al., like ourselves (P. Gray et al., unpub. data), were unable to grow ABV in cell lines of mammalian origin, such as Vero cells or MDCK cells in which BDV grows routinely. This research finding suggests that ABV may be unable to infect mammals.

Bornavirus has a nonsegmented negative strand genome. It encodes at least 6 proteins: N, X, P, M, G ,and L. The N or nucleoprotein interacts with the viral RNA and accumulates in the nucleus during the life cycle of the virus (*21*,*22*). The nucleoprotein of BDV exists in 2 isoforms of 40 and 38 kDa (*23*,*24*). P40 is primarily nuclear, and P38 is primarily cytoplasmic. Both isoforms can bind to the viral phosphoprotein. The immunofluorescent staining pattern observed with ABV-infected DEFs, which showed a punctate nuclear staining combined with a more diffuse cytoplasmic staining, is thus compatible with the known properties of the BDV nucleoprotein. Of 8 isolates reported here, 7 were of genotype 4 and 1 was of genotype 1. This finding may suggest that genotype 4 is a more pathogenic type associated with disease, or it may simply be the predominant strain circulating in Texas.

Rinder et al (*14*) reported on 6 isolates from Germany, 4 of which were genotype 4 and the others were genotype 2. This finding supports the suggestion that genotype 4 may be predominant worldwide and possibly more virulent than other genotypes. The experimental infection of 2 Patagonian conures with cultured virus that resulted in clinical PDD 66 days postinfection fulfills Koch’s postulates. PCR and sequencing of the amplified product demonstrated large amounts of ABV4 in the brains of the challenged birds. The birds did seroconvert for anti-N antibodies at 33 days, whereas fecal shedding was not detected until days 60–62. This finding is in contrast to observations on naturally infected birds in which fecal shedding may precede seroconversion by many months (*25*). ABV RNA was detected by RT-PCR after a minimum of 3 passages in DEF primary cell culture subsequent to inoculation with brain tissue from all 8 necropsy-confirmed PDD-positive birds. PCR detection in the brain tissue and ready isolation of the virus from freshly harvested brain tissue are compatible with the concept that PDD originates as a viral encephalitis (*11*).

Gancz et al. (*26*) have induced PDD in cockatiels (*Nymphicus hollandicus*) after inoculation of brain homogenates from PDD-affected, ABV-positive birds. Although the findings of Gancz et al. support our results and are in line with previous findings (*27*), interpretation of their results is difficult because of evidence for an autoimmune component in PDD similar to that which occurs in Guillain Barré syndrome (*28*). We also have detected autoantibodies to myelin basic protein and other nervous system autoantigens in PDD cases, suggesting that in this study, the brain homogenate may have contributed to the abnormalities observed. (*25*). The known pathogenesis of mammalian bornavirus infections fits well with the causative role of ABV in PDD. Both PDD and mammalian Borna disease share many attributes, including a viral encephalitis and polyneuritis with selective destruction of Purkinje cells, lymphocyte infiltration, and dysfunction of the central, peripheral, and autonomic nervous systems (*29*–*31*).

In conclusion, the results reported here together with previous findings confirm unequivocally that the long-sought cause of proventricular dilatation disease is indeed avian bornavirus. Investigations into this virus and the complex disease that it causes may provide useful insights into the pathogenesis of mammalian Borna disease. The origin and epidemiology, as well and prevention and treatment, of this infection remain to be elucidated.
