# Erratum—Vol. 16, No. 3

**DOI:** 10.3201/eid1604.C31604

**Published:** 2010-04

**Keywords:** *Keywords: errata*, *erratum*, *correction*

The author list for the article Use of Avian Bornavirus Isolates to Induce Proventricular Dilatation Disease in Conures (P. Gray et al.) omitted W. Ian Lipkin. The article has been corrected online http://wwwnc.cdc.gov/eid/article/16/3/09-1257_article.htm.

